# High *SEC61G* expression predicts poor prognosis in patients with Head and Neck Squamous Cell Carcinomas

**DOI:** 10.7150/jca.51467

**Published:** 2021-05-05

**Authors:** Leifeng Liang, Qingwen Huang, Mei Gan, Liujun Jiang, Haolin Yan, Zhan Lin, Haisheng Zhu, Rensheng Wang, Kai Hu

**Affiliations:** 1 Department of Oncology, The Sixth Affiliated Hospital of Guangxi Medical University, Yulin, Guangxi, China.; 2 Department of Pathology, The Sixth Affiliated Hospital of Guangxi Medical University, Yulin, Guangxi, China.; 3 Department of Radiation Oncology, The First Affiliated Hospital of Guangxi Medical University, Nanning, Guangxi, China.

**Keywords:** SEC61G, prognosis, biomarker, head and neck squamous cell carcinoma

## Abstract

**Background:** Overexpression of the membrane protein SEC61 translocon gamma subunit (SEC61G) has been observed in a variety of cancers; however, its role in head and neck squamous cell carcinomas (HNSCC) is unknown. This study aimed to elucidate the relationship between *SEC61G* and HNSCC based on data from The Cancer Genome Atlas (TCGA) database.

**Methods:** Data for HNSCC patients were collected from TCGA and the expression level of *SEC61G* was compared between paired HNSCC and normal tissues using the Wilcoxon rank-sum test. The relationship between clinicopathologic features and *SEC61G* expression was also analyzed using the Wilcoxon rank-sum test and logistic regression. Receiver operating characteristic (ROC) curves were generated to evaluate the value of *SEC61G* as a binary classifier using the area under the curve (AUC value). The association of clinicopathologic characteristics with prognosis in HNSCC patients was assessed using Cox regression and the Kaplan-Meier methods. A nomogram, based on Cox multivariate analysis, was used to predict the impact of *SEC61G* on prognosis. Functional enrichment analysis was performed to determine the hallmark pathways associated with differentially expressed genes in HNSCC patients exhibiting high and low *SEC61G* expression.

**Results:** The expression of *SEC61G* was significantly elevated in HNSCC tissues compared to normal tissues (P < 0.001). The high expression of *SEC61G* was significantly correlated with the T stage, M stage, clinical stage, *TP53* mutation status, *PIK3CA* mutation status, primary therapy outcome, and cervical lymph node dissection (all P < 0.05). Meanwhile, ROC curves suggested the significant diagnostic ability of *SEC61G* for HNSCC (AUC = 0.923). Kaplan-Meier survival analysis showed that patients with HNSCC characterized by high *SEC61G* expression had a poorer prognosis than patients with low *SEC61G* expression (hazard ratio = 1.95, 95% confidence interval 1.48-2.56, P < 0.001). Univariate and multivariate analyses revealed that *SEC61G* was independently associated with overall survival (P = 0.027). Functional annotations indicated that SEC61G is involved in pathways related to translation and regulation of SLITs/ROBOs expression, SRP-dependent co-translational protein targeting to the membrane, nonsense-mediated decay, oxidative phosphorylation, and Parkinson's disease.

**Conclusion:** *SEC61G* plays a vital role in HNSCC progression and prognosis; it may, therefore, serve as an effective biomarker for the prediction of patient survival.

## Introduction

HNSCC, including oral, oropharyngeal, and laryngeal cancers, is among the top 10 most common diseases worldwide, and with more than 65,000 new cases and approximately 15,000 deaths each year in the United States alone 1. Despite active comprehensive treatment and recent advances in therapeutic options, the prognosis for HNSCC patients remains unsatisfactory, with a 5-year survival rate estimated at 65%, even lower in the advanced diseases 1. The low survival rates associated with HNSCC are partly due to the failure of early diagnosis, which is primarily attributed to the lack of appropriate screening and diagnostic biomarkers 2, 3. Although various biomarkers have been applied to HNSCC, such as human papillomavirus status and programmed cell death protein 1 4, their reliability remains controversial. Thus, identification of new biomarkers related to tumor stage and prognosis is exceedingly important to facilitate early diagnosis, prognosis evaluation, and treatment of HNSCC.

SEC61G is one of three subunits comprising the SEC61 membrane protein complex 5, which represents the central module of the protein translocation apparatus in the endoplasmic reticulum membrane 6. SEC61G participates in protein folding, modification, translocation, and the unfolded protein response, particularly under the conditions of hypoxia and nutrient deprivation in tumor microenvironment 7-9. SEC61G was also found to be overexpressed in gastric cancer 10, breast cancer 11, and glioblastoma 12. However, the association of SEC61G with HNSCC has not yet been characterized.

In this study, we sought to demonstrate the correlation between *SEC61G* and HNSCC and to analyze the prognostic role of *SEC61G* in HNSCC based on RNA-sequencing (RNA-seq) data from TCGA. Moreover, we analyzed the expression levels of *SEC61G* in HNSCC and normal tissues and determined the correlation between *SEC61G* expression and patient prognosis in terms of overall survival (OS). Further, we performed prognosis and clinical correlations to explore the potential diagnostic and prognostic value of *SEC61G*; while its biological significance was defined using enrichment analysis, molecular interaction network analysis and immune infiltration correlation analysis. Taken together, our study suggests that *SEC61G* represents a significant independent predictor for HNSCC.

## Materials and methods

### RNA-seq data source

A total of 501 HNSCC cases with gene expression data (HTSeq-FPKM) were collected from TCGA. Samples with RNA-seq data that lacked corresponding clinical data were excluded from the analysis. Level-3 HTSeq-FPKM data were transformed into transcripts per million reads (TPM) for subsequent analyses. After filtering, the TPM data of 500 HNSCC patients were further analyzed. Gene expression data were divided into a high expression group and a low expression group according to the median *SEC61G* expression level. This study met the publication guidelines stated by TCGA (https://cancer genome.nih.gov/publications/publication guidelines). All data used were acquired from TCGA, and hence, ethical approval and informed consent of the patients were not required.

### Pathological sample collection

Samples were collected for paired tumor and normal tissues from 60 patients with HNSCC diagnosed between January 2015 and December 2015. All patient-derived information and specimens were collected and archived under the protocols approved by the Institutional Review Boards of The Sixth Affiliated Hospital of Guangxi Medical University (Approve No. YLSY-IRB-CR-2020087). This study was performed in accordance with the principles of the Declaration of Helsinki.

### Immunohistochemistry study

Immunohistochemistry (IHC) was performed on formalin-fixed, paraffin-embedded tissue sections. SEC61G was detected with the rabbit anti-SEC61G polyclonal antibody (11147-2-AP, Proteintech Group Inc., Wuhan Sanying Biotechnology Development Co. Ltd.). Paraffin slices were dewaxed in xylene. A gradient alcohol dehydration process was then performed consisting of 100%, 95%, 80%, and 70% ethanol (2 min each). The tissues were subsequently rinsed with distilled water twice for 5 min each, and with phosphate-buffered saline (PBS) thrice, for 5 min each. Next, pressure cooker-mediated antigen retrieval was performed in EDTA buffer, pH 9.0 (Fuzhou Maixin Biotechnology Development Co. Ltd.) for 30 min. Sections were incubated with a 1:100 dilution of anti-SEC61G antibody for 30 min at 37 °C and subsequently incubated with enzyme-labeled anti-mouse/rabbit lgG polymer (Fuzhou Maixin Biotechnology Development Co. Ltd.) for 30 min at 25 °C. After rinsing with PBS three times for 5 min each, the sections were incubated with DAB chromogenic reagent (Fuzhou Maixin Biotechnology Development Co., Ltd.) for 5 min, followed by counterstaining with Mayer's hematoxylin, dehydrating, and mounting.

Two independent single-blinded pathologists assessed the sections and applied a semiquantitative scoring system 13 to evaluate staining intensity (0, no staining; 1+, weak staining; 2+, moderate staining; 3+, strong staining) and the percentage of stained cells (0, < 5%; 1, 5%-25%; 2, 26%-50%; 3, 51%-75%; and 4, > 75%). The staining intensity scores were multiplied by the percentage of positive cells to generate the immunoreactivity score of each case 14. All cases were sorted into two groups according to the immunoreactivity score. Positive of SEC61G was defined as detectable immunoreactions in the cytoplasmic or membrane with an immunoreactivity score of ≥ 1.

### Enrichment analysis

The Search Tool for the Retrieval of Interacting Genes (STRING; http://strin g-db.org; version 10.0) online database was used to predict the protein-protein interaction network of *SEC61G* co-expressed genes and to analyze the functional interactions among proteins 15. An interaction with a combined score > 0.4 was considered statistically significant. The expression profiles (HTSeq-Counts) between the high SEC61G expression group and the low SEC61G expression group were compared to identify differentially expressed genes (DEG) using Wilcoxon rank-sum test in the R language-related software, DESeq2 (3.8). 16. Differences with a |log fold change (FC)|>1.5 and adjusted P-value < 0.05 were considered as threshold values for identifying DEGs 17-20. Clusterprofiler 21 software 3.6.0 was applied to perform Gene Ontology (GO) function enrichment analyses on the DEGs identified between the high and low *SEC61G* expression groups.

### Gene set enrichment analysis (GSEA)

GSEA 22 is a computational method that determines whether a defined set of genes exhibits statistically significant concordant differences between two biological states. In this study, GSEA was performed with the R package ClusterProfiler 21 to elucidate the significant function and pathway differences between the high and low *SEC61G* expression groups. Each analysis procedure was repeated 1000 times. A function or pathway term withadjusted P-value < 0.05, and false discovery rate (FDR) < 0.25 was considered to statistically significant enrichment 23-25.

### Immune infiltration analysis by single-sample GSEA (ssGSEA)

Immune infiltration analysis of HNSCC samples was performed by the ssGSEA method using the GSVA package in R (http://www.biocondutor.org/package/release/bioc/html/GSVA.html) for 24 types of immune cells, including neutrophils, mast cells, eosinophils, macrophages, natural killer (NK) cells, CD56^dim^ NK cells, CD56^bright^ NK cells, dendritic cells (DCs), immature DCs (iDCs), activated DCs (aDCs), plasmacytoid DCs (pDCs), T cells, CD8+ T cells, T helper (Th) cells, Th1 cells, Th2 cells, Th17 cells, T follicular helper cells, regulatory T cells (Treg), central memory T cells (Tcm), effector memory T cells (Tem), gamma delta T cells (Tgd), cytotoxic cells, and B cells. Based on the reported signature genes for the 24 types of immunocytes 26, the relative enrichment score of each was quantified from the gene expression profile for each tumor sample. Spearman's correlation coefficient analysis was performed to identify relationships HNSCC and each immune cell subset. Moreover, immune cell infiltration was investigated between the high and low *SEC61G* expression groups using the Wilcoxon rank-sum test.

### Construction and evaluation of the nomogram

To individualize the predicted survival probability for l, 3, and 5 years, a nomogram was constructed based on the results of the multivariate analysis. The RMS R package was used to generate a nomogram including clinical characteristics significantly associated with *SEC61G* and calibration plots. Calibration and discrimination are the most used methods for evaluating the performance of models. In this study, the calibration curves were graphically assessed by mapping the nomogram-predicted probabilities against the observed rates, and the 45-degree line represented the best predictive values. The concordance index (C-index) was used to determine the discrimination of the nomogram, which was calculated by a bootstrap approach with 1000 resamples. In addition, the predictive accuracies of the nomogram and individual prognostic factors were compared using the C-index and ROC analyses. All statistical tests were two-tailed with the statistical significance level set at 0.05.

### Statistical analysis

All statistical analyses and plots were conducted using R (Version 3.6.3). Wilcoxon rank-sum test and Wilcoxon rank signed test were used to analyze the expression of *SEC61G* in non-paired samples and paired samples, respectively. Kruskal-Wallis test, Wilcoxon rank-sum test, and logistic regression evaluated relationships between clinical-pathologic features and *SEC61G* expression. Furthermore, ROC analysis and the frequently-used method for binary assessment were performed using the pROC package 27 to assess the effectiveness of *SEC61G* expression to discriminate HNSCC from normal samples. The computed AUC value ranging from 0.5 to 0.1 indicates the discriminative potential from 50% to 100%. The prognostic data was obtained from Cell 28, while Cox regression analyses and the Kaplan-Meier method were used to evaluate prognostic factors. In all tests, P-value < 0.05 was considered statistically significant.

## Results

### Elevated expression of *SEC61G* in HNSCC

**Figure 1:**
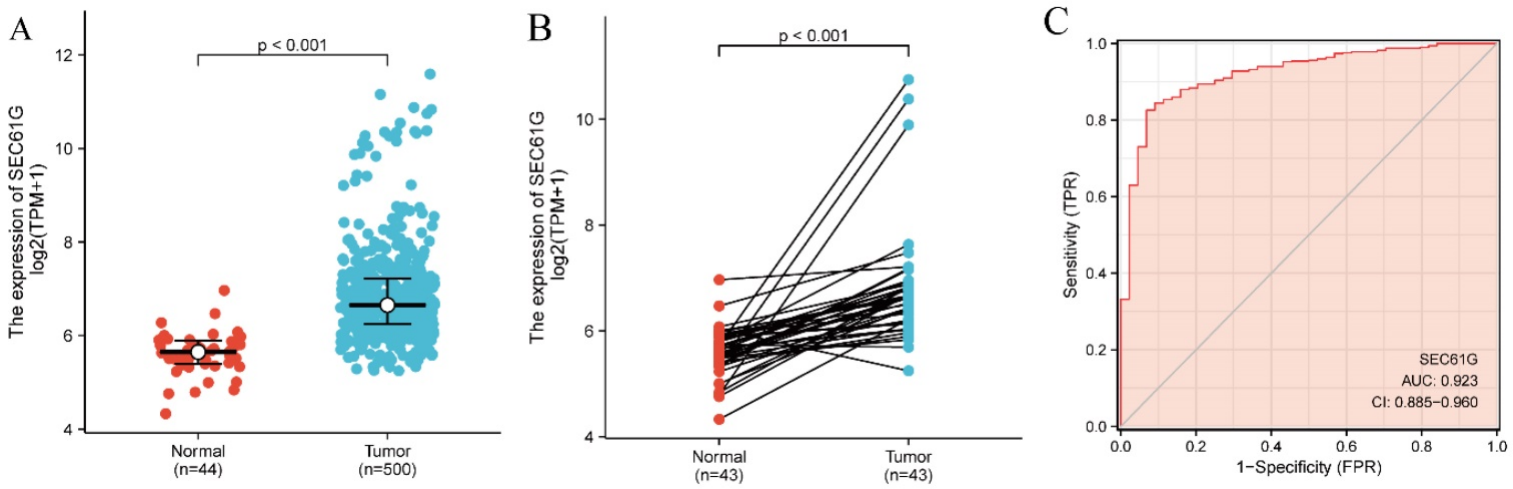
***SEC61G* expression between cancer and normal tissues in HNSCC patients** (A) *SEC61G* expression levels in HNSCC and matched normal tissues. (B) *SEC61G* expression levels in HNSCC and matched normal tissues. (C) ROC analysis of *SEC61G* shows promising discrimination power between tumor and normal tissues.

The *SEC61G* expression levels in 500 tumor tissues were substantially and significantly higher than those in 44 normal tissues (P < 0.001; Figure 1A) and were also higher in the 43 tumor tissues compared with paired normal tissues (P < 0.001; Figure 1B). In addition, *SEC61G* expression showed promising discriminative power with an AUC value of 0.923 to identify tumors from normal tissue (Figure 1C).

**Figure 2:**
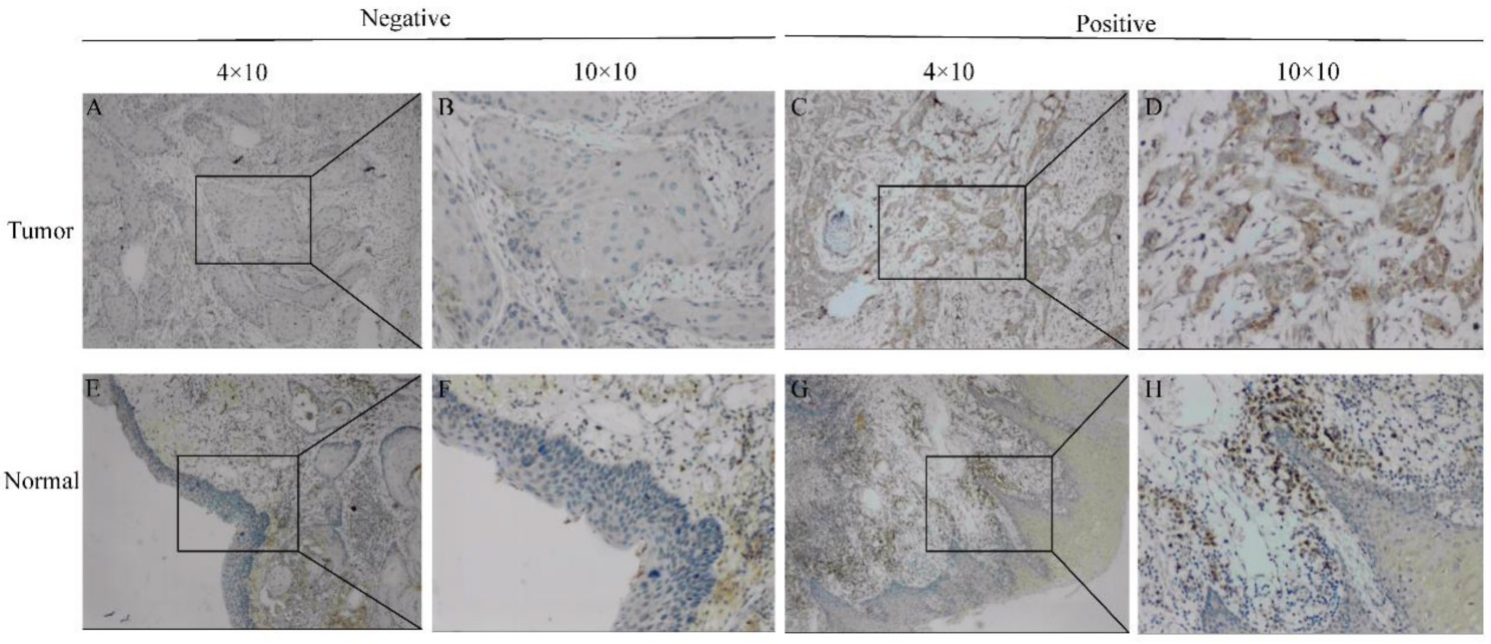
** Representative images of SEC61G expression in tongue cancer tissues and their normal controls**. (A, B) Negative or (C, D) positive staining for SEC61G in tongue cancer tissues. (E, F) Negative or (G, H) positive staining for SEC61G in normal tissues. Original magnifications 40× and 100× (inset panels).

To further determine the significance of SEC61G expression, IHC staining was performed for a cohort comprising 60 cases of primary HNSCC paired with noncancerous tissue. There were 42 males and 18 females with a mean age of 59 years (range 29-80 years) involved in our HNSCC cohort. According to the SEC61G IHC staining, 78.3% (47/60) of normal tissues were negative while 63.3% (38/60) of HNSCC tissues were positive. Representative images are presented in Figure 2.

### Associations between *SEC61G* expression and clinicopathologic variables

**Figure 3:**
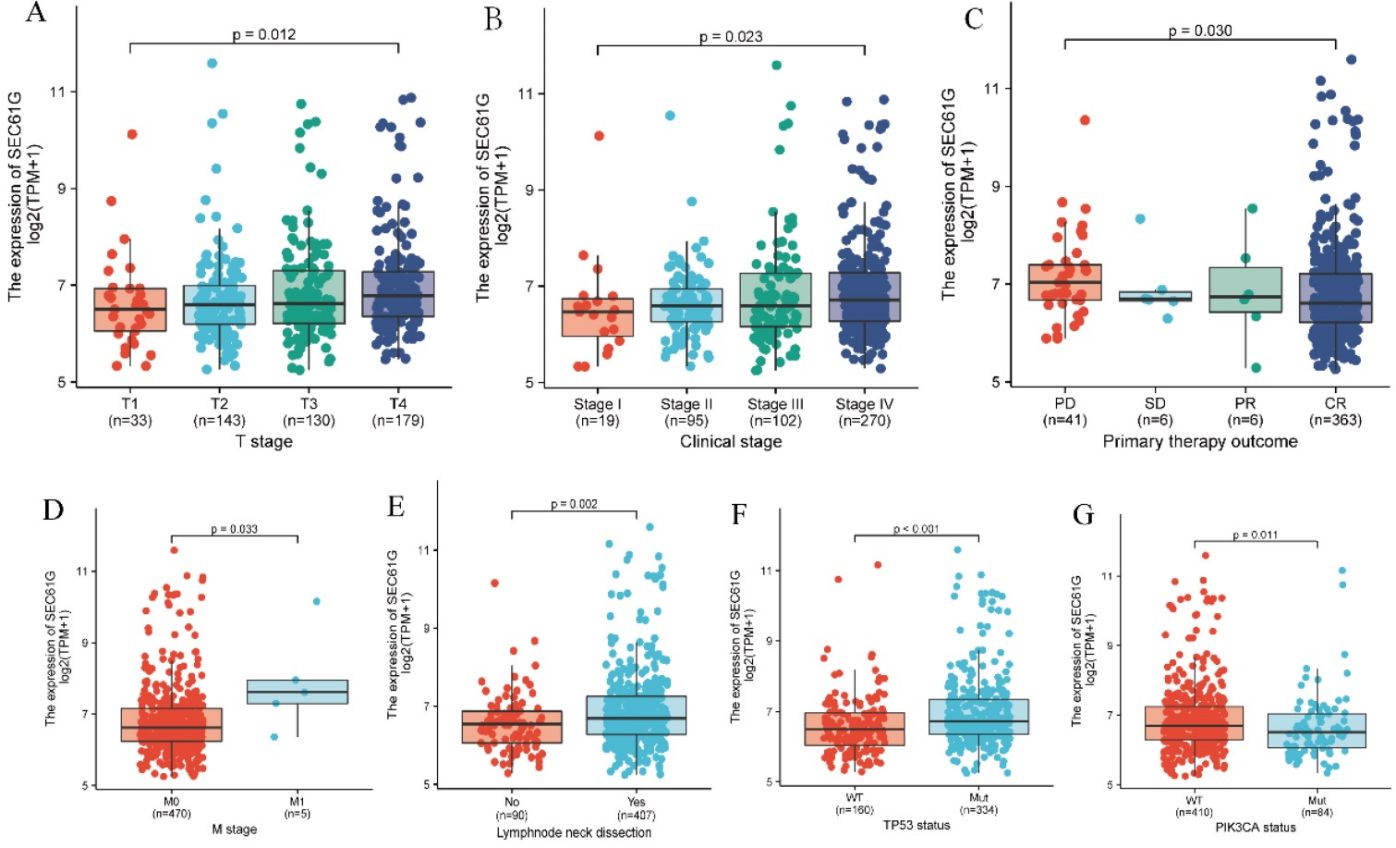
** Association of *SEC61G* expression with clinicopathologic characteristics.** (A) T stage; (B) clinical stage; (C) primary therapy outcome; (D) M stage; (E) lymph node neck dissection; (F) *TP53* mutation status; (G) *PIK3CA* mutation status.

The Kruskal-Wallis rank-sum test showed that the level of *SEC61G* was significantly correlated with T stage (P = 0.012), clinical stage (P = 0.023), and primary therapy outcome (P = 0.030; Figure 3A-C). Meanwhile, results of the Wilcoxon rank-sum test found that *SEC61G* expression level was significantly correlated with M stage (P = 0.033), cervical lymph node dissection (P = 0.002), *TP53* mutation status (P < 0.001), and *PIK3CA* mutation status (P = 0.011; Figure 3D-G).

**Figure 4:**
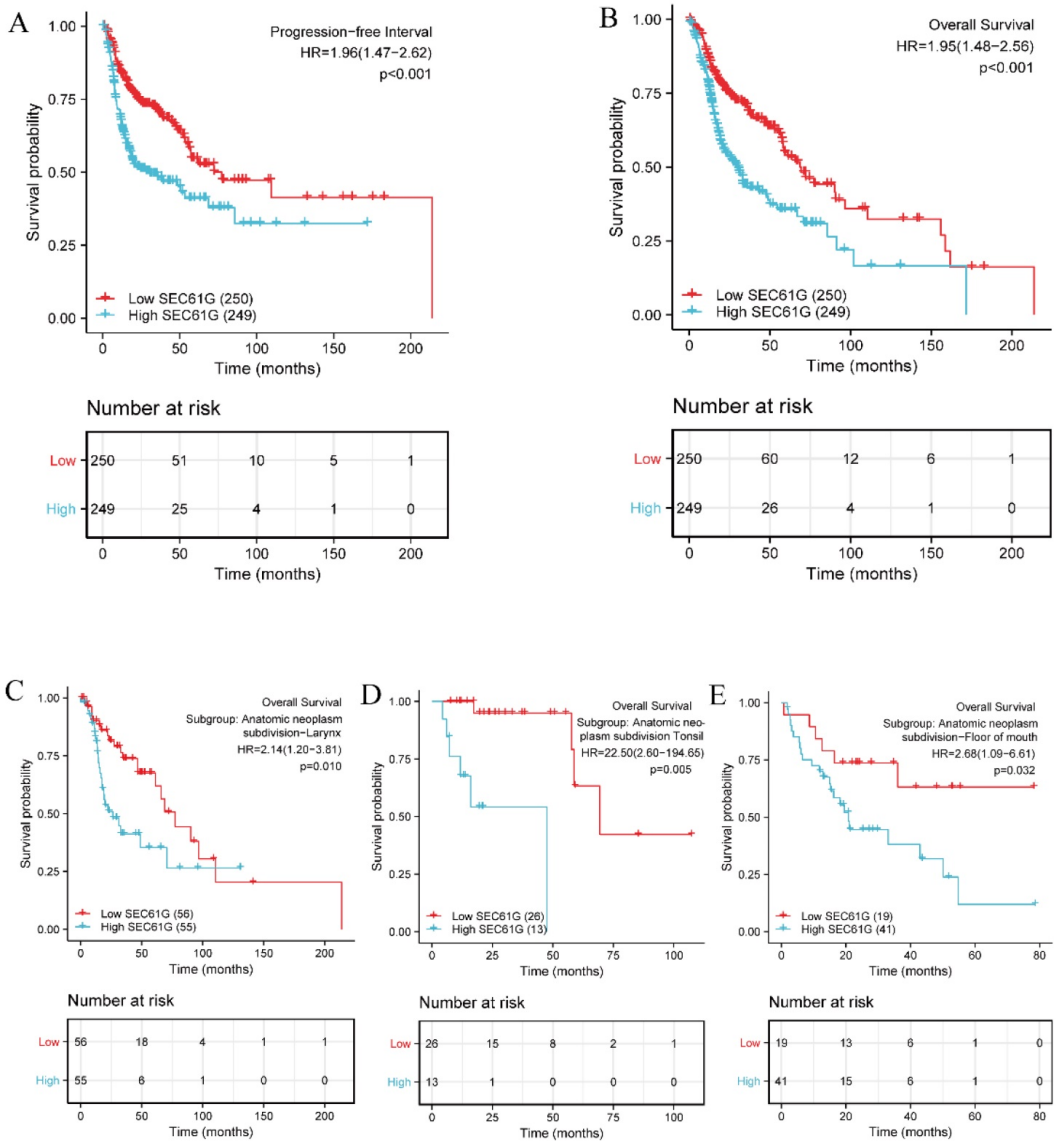
** Kaplan-Meier survival curves comparing the high and low expression of *SEC61G* in HNSCC patients.** (A) Progression-free interval. (B) Overall survival. (C-E) Overall survival for subgroup analyses in different HNSCC anatomical sites: larynx (C), tonsil (D), and floor of mouth cancer (E).

Moreover, Kaplan-Meier survival analysis showed that high *SEC61G* expression was more strongly associated with a poor prognosis than low *SEC61G* expression (Figure 4A and Figure 4B, P < 0.001). Subgroup analyses further demonstrated that *SEC61G* expression correlated with OS in different HNSCC anatomic sites, including the larynx (P = 0.010, Figure 4C), tonsil (P = 0.005, Figure 4D) and floor of mouth (P = 0.032, Figure 4E) cancers.

### Impact of high *SEC61G* expression on the prognosis of HNSCC patients with different clinicopathological status

To better understand the relevance and mechanisms of *SEC61G* expression in HNSCC, we investigated the relationship between *SEC61G* expression and clinical characteristics of HNSCC patients by univariate Cox analysis. Other clinicopathologic variables that correlated with poor survival included the M stage, radiation therapy, primary therapy outcome, lymphovascular invasion, and *TP53* mutation status. To further explore factors associated with survival, multivariate Cox regression analysis was performed and found that high *SEC61G* expression remained an independent factor associated with poor OS, along with radiation therapy, primary therapy outcome, and lymphovascular invasion (Table 1).

### Elevated expressions of SEC61G predict poor prognosis in different cancer stages

**Figure 5:**
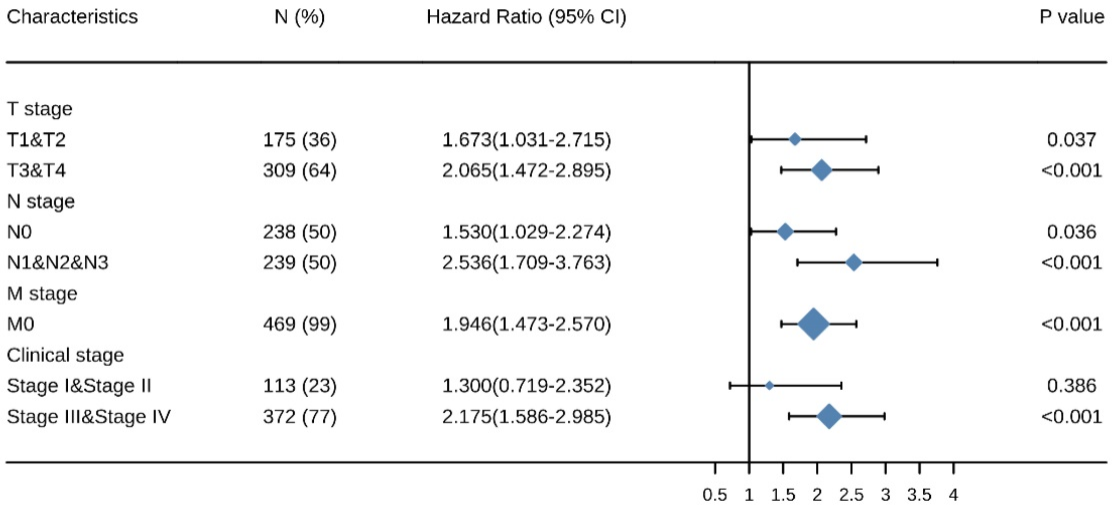
Multivariate survival analysis of overall survival probabilities concerning *SEC61G* expression in patients of different subgroups according to cancer stage.

Kaplan-Meier survival analysis showed that HNSCC patients with high *SEC61G* expression had a worse prognosis than patients with low SEC61G expression in the different cancer stage categories: T (T1 & T2, P = 0.037, T3 & T4, P < 0.001), N (N0, P = 0.036, N1 & N2 & N3, P < 0.001), M (M0, P < 0.001), and clinical stage (III & IV, P < 0.001) (Figure 5). These results suggest that the *SEC61G* expression level can impact prognosis in HNSCC patients at different pathological stages.

### Construction and validation of a nomogram based on *SEC61G* expression

**Figure 6:**
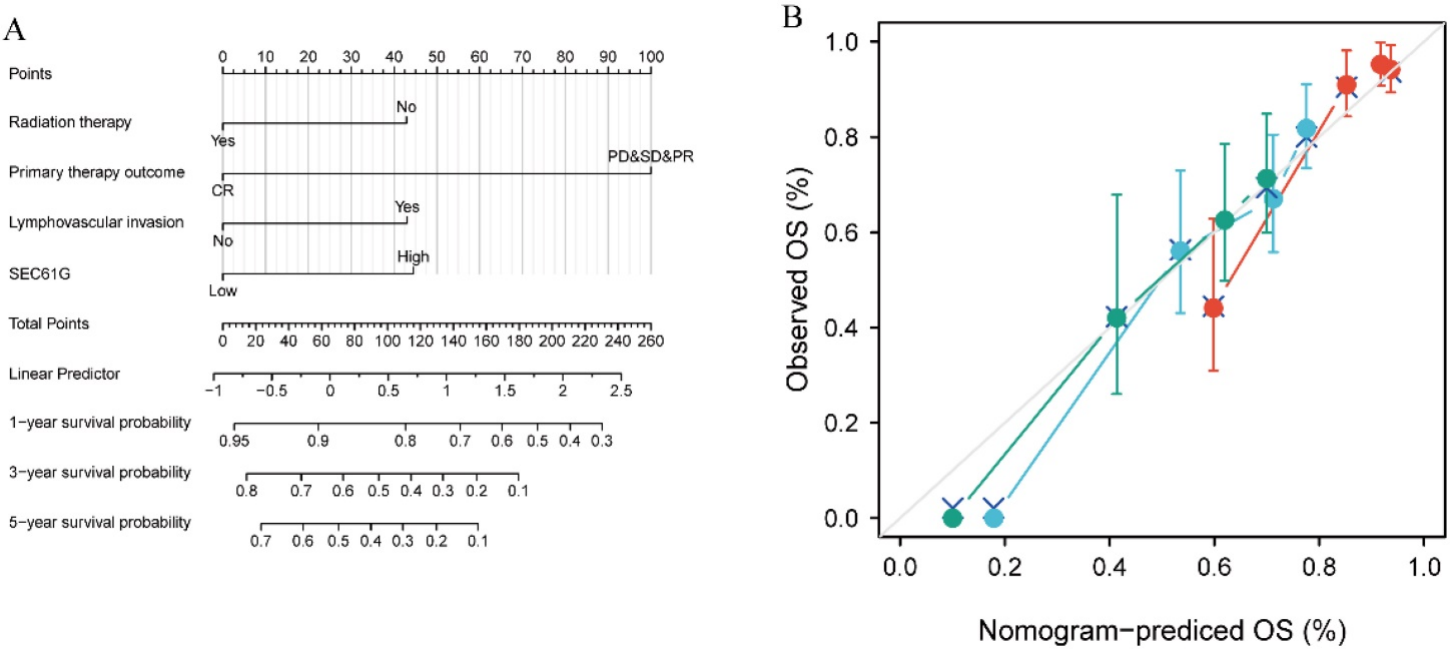
** Relationship between *SEC61G* and other clinical factors with overall survival (OS).** (A) Nomogram for predicting the probability of 1-, 3-, and 5-year OS for HNSCC patients. (B) Calibration plot of the nomogram for predicting the OS likelihood.

To provide clinicians with a quantitative approach for predicting the prognosis of HNSCC patients, a nomogram was constructed that integrated the clinical characteristics determined to be independently associated with survival via multivariate analysis (radiation therapy, primary therapy outcome, lymphovascular invasion, and *SEC61G*; Figure 6A). A point scale was used to assign these variables to the nomogram based on multivariate Cox analysis: a straight line was used to determine the points for the variables, and the sum of the points awarded to each variable was rescaled on a range from 0 to 100. The positions of the variables were accumulated and recorded as the total points. The probability of HNSCC patients' survival at 1, 3, and 5 years was determined by drawing vertical lines from the total point axis downward to the outcome axis. Using survival ROC package, the time-dependent ROC for *SEC61G* to 1-year, 3-year, and 5-year OS was analyzed (Supplementary Figure S1A), as well as that for the established nomogram prognostic model (Supplementary Figure S1B). According to the median value (-0.292), the score of the nomogram prognostic model was divided into high and low groups. Kaplan-Meier survival analysis revealed that high nomogram scores significantly correlated with a worse prognosis than low nomogram score expression (P < 0.001; Supplementary Figure S2).

Moreover, within the nomogram, *SEC61G* expression was found to contribute the most extreme data points (ranging from 0 to 100) compared with the other clinical variables, which was consistent with the results of multivariate Cox regression. The C-index of the nomogram was 0.681 with 1000 bootstrap replicates (95% confidence interval: 0.658-0.703). The bias-corrected line in the calibration plot was close to the ideal curve (i.e., the 45-degree line), indicating good agreement between the predicted and observed values (Figure 6B). Overall, the nomogram was found to be a superior model for predicting long-term survival in HNSCC patients than individual prognostic factors.

### Identification of DEGs between the high and low *SEC61G* expression groups

**Figure 7:**
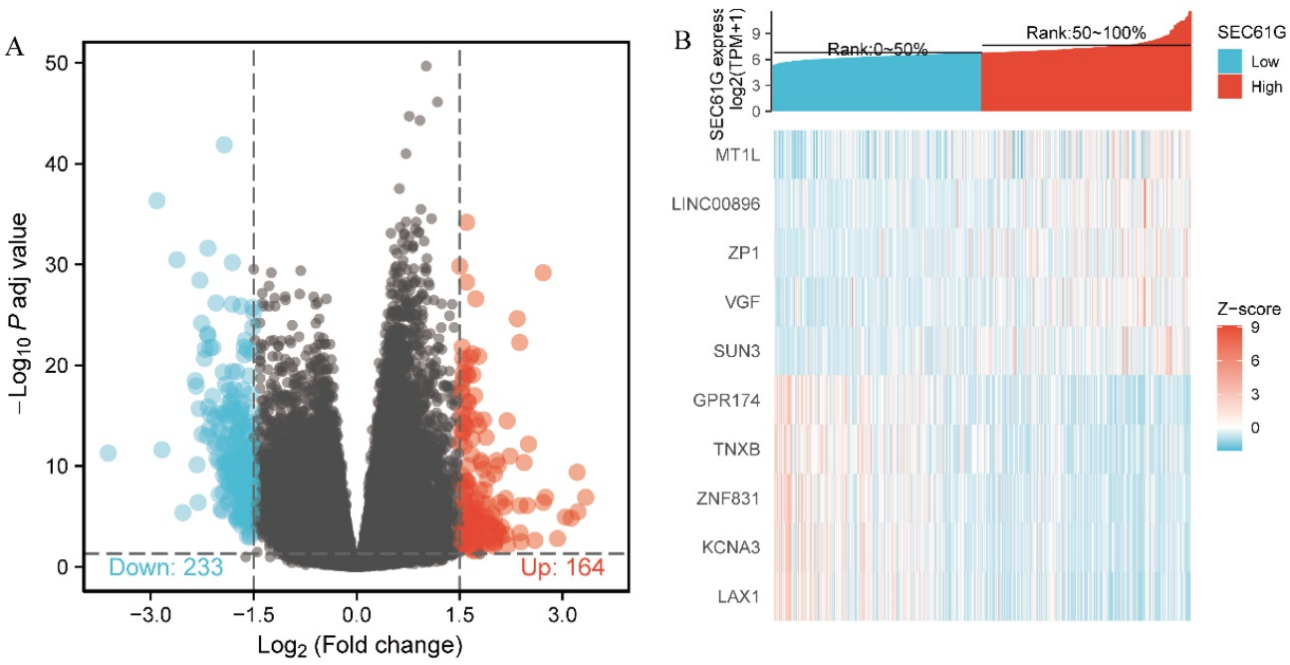
** Differentially expressed genes between patients with high and low *SEC61G* expression.** (A) Volcano plot of differentially expressed genes between the high and low *SEC61G* expression groups. Normalized expression levels are shown in descending order from green to red. (B) Heatmap of the top ten significant differentially expressed genes between the high and low *SEC61G* expression groups. Green and red dots represent downregulated and upregulated genes, respectively.

The data from TCGA were analyzed using the DSEeq2 package in R (|logFC|>1.5, adjusted P-value < 0.05) and 397 DEGs were identified between the groups with high and low *SEC61G* expression, including 164 upregulated and 233 downregulated genes in the high expression group (Figure 7).

### Functional annotation and predicted signaling pathways

**Figure 8:**
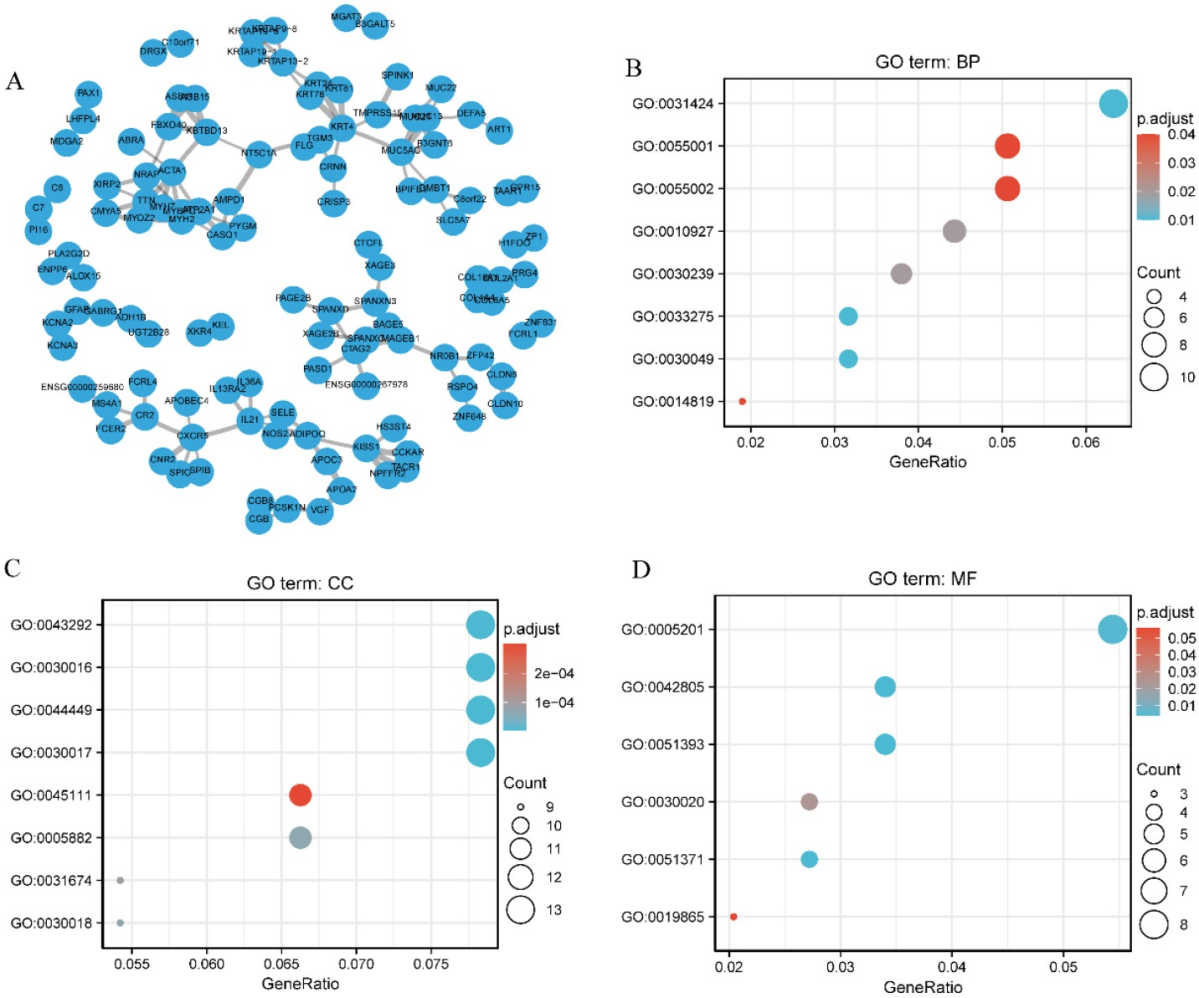
** Protein-protein interaction network and functional enrichment analysis.** (A) Protein-protein interaction network of *SEC61G* and its co-expressed genes. (B) Enriched GO terms in the “biological process” category. (C) Enriched GO terms in the “cellular component” category. (D) Enriched GO terms in the “molecular function” category. The x-axis represents the proportion of differentially expressed genes (DEGs) and the y-axis represents different categories. Blue and red tones represent adjusted P values from 0.0 to 0.05, respectively, and different circle sizes represent the number of DEGs.

A network of *SEC61G* and its potential co-expressed genes in *SEC61G* are shown in Figure 8A. To better understand the functional implication of *SEC61G* in HNSCC from the 397 DEGs identified between the high and low expression groups, GO enrichment analysis was performed using the ClusterProfile package. Sixteen enriched terms were identified in the GO “biological process” category, including keratinization, muscle cell development, and cellular component assembly involved in morphogenesis (Figure 8B). These results suggest a link between the aberrant expression of *SEC61G* and keratinization. Meanwhile, 29 GO terms within the “cellular component” category were associated with contractile fibers (Figure 8C). Furthermore, the “molecular function” category revealed significant enrichment in GO terms related to the extracellular matrix structural constituents (Figure 8D).

### SEC61G-related signaling pathways based on GSEA

**Figure 9:**
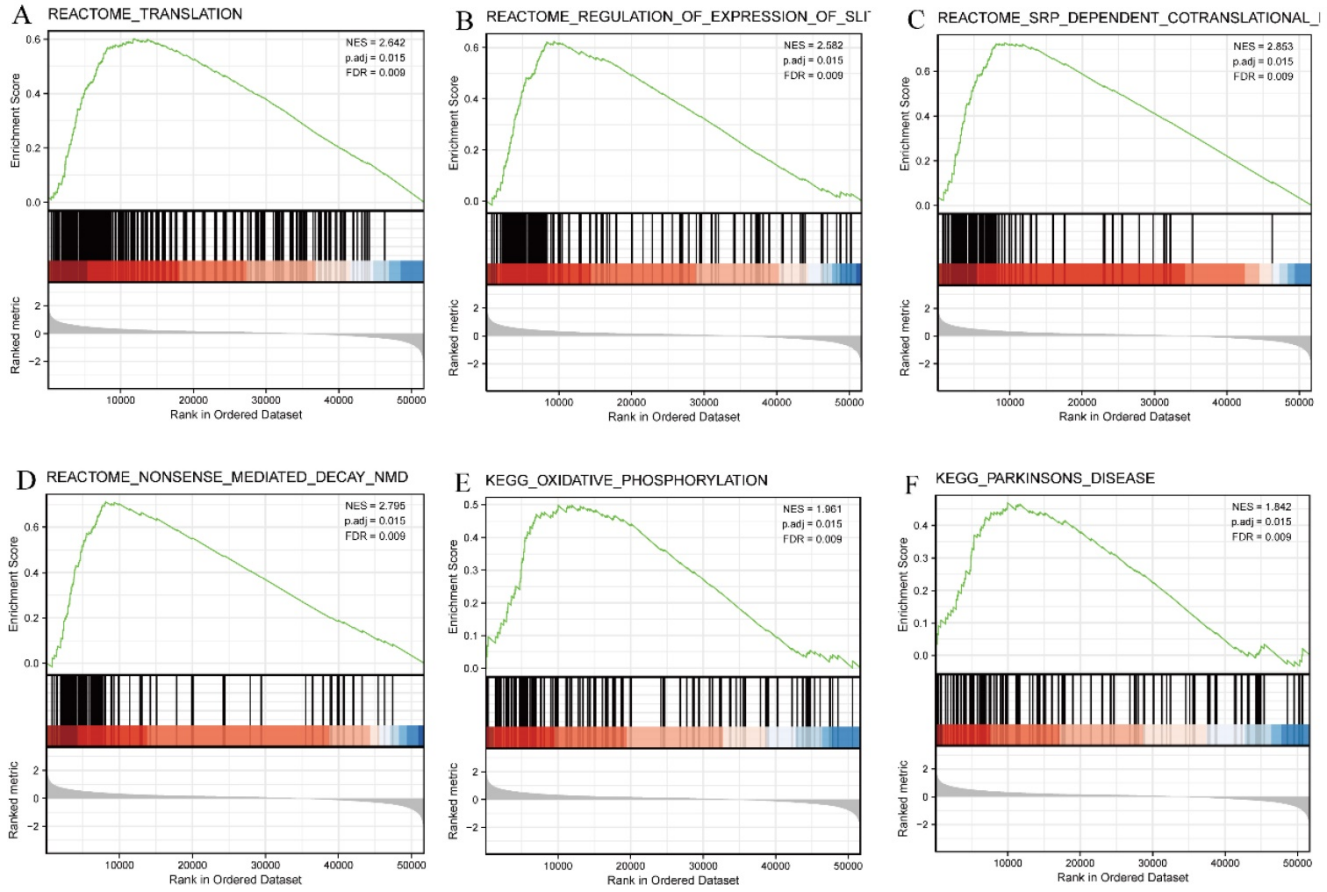
**Enrichment plots from GSEA.** Several pathways were differentially enriched in HNSCC patients according to high and low *SEC61G* expression. (A) Translation. (B) Regulation of the SLITs/ROBOs pathway expression. (C) SRP-dependent co-translational protein targeting the membrane. (D) Nonsense-mediated decay pathway. (E) Oxidative phosphorylation. (F) Parkinson disease. ES, enrichment score; NES, normalized enrichment score; ADJ p-Val, adjusted P-value; FDR, false discovery rate.

GSEA was then used to identify signaling pathways associated with HNSCC between the high and low *SEC61G* expression groups, based on significant differences (adjusted P-value < 0.05, FDR < 0.25) in the enrichment of MSigDB Collection (c2.all.v7.0.symbols.gmt [Curated]). Six pathways, including translation, regulation of expression of the SLITs/ROBOs pathway, SRP-dependent co-translational protein targeting the membrane, nonsense-mediated decay pathway, oxidative phosphorylation, and Parkinson's disease, were identified as significantly different between the two groups (Figure 9).

### Correlation between *SEC61G* expression and immune infiltration

**Figure 10:**
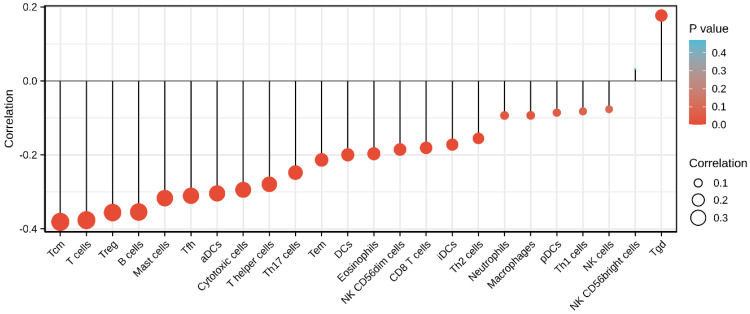
** Correlations between the relative abundance of 24 immune cells and *SEC61G* expression levels.** The size of the dots represents the absolute Spearman's correlation coefficient values.

Finally, we analyzed the correlation between the expression level (TPM) of *SEC61G* and immune cell enrichment (generated by ssGSEA) based on the Spearman correlation coefficient. *SEC61G* expression was negatively correlated with the abundance of Tcm, T cells, Tregs, B cells, mast cells, Tfh, cytotoxic cells, Th cells, Th17 cells, Tem, DCs, eosinophils, NK CD56^dim^, CD8 T cells, and iDCs, and was positively correlated with the abundance of NK CD56^bright^ cells and Tgd. (Figure 10).

## Discussion

An effective prognostic biomarker provides important information regarding cancer aggressiveness and/or the clinical outcome of a specific patient in the absence of treatment. However, they are also crucial components in personalized medicine and precision medicine, as they can prevent undertreatment or overtreatment. Previous studies have also revealed that *SEC61G* participates in the regulation of multiple disease states including diabetes, neurodegeneration, and cancer 7. More recently, SEC61G was found to be overexpressed in gastric cancer, breast cancer, and glioblastoma 10-12. However, little is known regarding its prognostic value in HNSCC. Our results consistently demonstrate that *SEC61G* expression serves as a robust predictor of HNSCC clinical outcome.

In the current study, we performed a bioinformatics analysis to assess the prognostic value of SEC61G in HNSCC using high-throughput RNA-seq data obtained from TCGA. We observed that SEC61G was more highly expressed in tumor tissues compared to normal samples. Moreover, overexpression of SEC61G in cancer tissues correlated with poor clinicopathologic factors, suggesting that *SEC61G* functioned as an oncogene. Previous *in vitro* functional assays revealed that *SEC61G* knockdown inhibited non-small-cell lung cancer cell proliferation and invasion while promoted apoptosis 29. In addition, compared with low *SEC61G* expression, high expression was significantly correlated with a poor OS in HNSCC patients. Similarly, Liu et al. reported that *SEC61G* expression may represent a potential prognostic marker for poor survival in glioblastoma patients 9. Hence, we conjectured that SEC61G may also serve as a biomarker for HNSCC.

To further investigate the biological function of SEC61G in HNSCC, we used TCGA data for GSEA and found that genes associated with regulation of the SLITs/ROBOs pathway were differentially enriched in the *SEC61G* high expression HNSCC phenotype. SLITs interact with ROBOs, the complexes of which subsequently play a considerable role in muscle cell formation, angiogenesis, cell migration, stem cell growth, organ development, and tumor formation by recruiting different adaptor molecules or proteins to activate a cascade of signaling pathways 30, 31. However, little is known how accurately SLIT binding to ROBO is communicated across the cell membrane 32. Meanwhile, increasing evidence suggests that SLITs can also bind to extracellular matrix molecules 33, 34. The mobile combinations of SLITs and ROBOs depend on different environments. Notably, P-cadherin was shown to be involved in regulating cell-cell adhesion by combining with ROBO3 in oral squamous cell carcinoma 35. In addition to weakening cell-cell adhesion, the assembly of cytoskeletal actin and dissolution of the extracellular matrix can be affected by SLIT/ROBO signaling to regulate cancer cell metastasis 36, 37. Overexpression of SLITs/ROBOs has also been observed in melanoma 38, gastric cancer 39, pancreatic cancer tissues and cell lines 40, as well as hepatocellular carcinoma 41, thereby demonstrating that SLITs/ROBOs signaling has a facilitating effect in certain cancers. It is, therefore, reasonable to conclude that blockade of SLITs/ROBOs signaling may potently inhibit tumor angiogenesis and effectively prevent malignant transformation for suppression of tumor growth and metastasis, as observed for colorectal carcinoma 42. However, studies have also reported that the expression of SLIT is downregulated, or not detected, in most tumors, including breast cancer 43, gastric cancer 44, lung cancer 45, liver cancer 46, esophageal cancer 47, among others, and is largely related to promoter hypermethylation, demonstrating the inhibitory effect of SLITs/ROBOs in these cancers 43-47. Meanwhile, in the current study, we found that SEC61G may exhibit crosstalk with pathways regulating the expression of SLITs/ROBOs. However, it is unclear whether SEC61G and regulation of the SLITs/ROBOs pathway have a synergistic effect or a complementary effect, and the detailed regulation network has not been reported. Moreover, our protein-protein interaction network revealed that SEC61G participates in sophisticated crosstalk with numerous other genes. Thus, further investigations are warranted to elucidate the detailed molecular mechanisms underlying these interactions. In addition, SEC61G expression is regulated by the long non-coding RNA LINC02418 29, suggesting that SEC61G may be involved in a more complex regulatory network.

The tumor microenvironment is regarded as a crucial interface mediating physiological reactions in cancer cells. Tumor infiltrating immune cells account for an indispensable component of the tumor microenvironment with their composition and distribution considered to be related to cancer prognosis. Given the crucial role of the tumor microenvironment in mediating cancer progression and tumor-infiltrating immune cells account for an indispensable component of the tumor microenvironment 48, 49, we sought to investigate the relationship between SEC61G and immune infiltration in HNSCC. As shown in figure 10, it shows that increased SEC61G expression was negatively correlated with the abundances of B cells, CD8+ T cells, and Tregs in HNSCC. Previous studies have revealed that low levels of infiltrating B cells and cytotoxic CD8+ T in tumor tissues were associated with poor prognosis of cancer patients including HNSCC 50-54. On the contrary, several other studies have reported that high levels of tumor-infiltrating Tregs were significantly associated with worse outcome in breast cancer 55, hepatocellular carcinoma 56, pancreatic ductal adenocarcinoma 57, lung cancer 58, gastric cancer 59, and ovarian cancer 60. But interestingly, in the context of HNSCC, patients with elevated Treg levels reportedly have a significantly better OS 61. We, therefore, inferred that *SEC61G* might affect the prognosis of patients by modulating immune infiltration in HNSCC.

Although our approaches can provide new insights into the correlation between SEC61G and HNSCC, certain limitations were noted in this study. First, only one dataset was assessed, which may cause sample bias. Second, to increase the credibility of the results, the sample size should be further expanded. Third, to improve the clinical application, additional clinical factors should be included. Fourth, further experimental verification is required to elucidate the biological functions of *SEC61G in vitro* and *in vivo*.

In summary, our study revealed the prognostic value of *SEC61G* in HNSCC for the first time. Our findings strongly suggest that *SEC61G* offers potential as a biomarker to predict the treatment outcome and prognosis in HNSCC patients. Further experimental validation is warranted, however, to elucidate the biological impact and underlying mechanism of *SEC61G*.

## Supplementary Material

Supplementary figures.

## Figures and Tables

**Table 1:** Association of clinicopathological characteristics with overall survival using univariate or multivariate Cox regression analysis

Characteristic	Sample (N)	Univariate analysis		Multivariate analysis	
		HR (95% CI)	*P*-value	HR (95% CI)	*P*- value**
T stage (T3 & T4 vs. T1 & T2)	484	1.230 (0.921-1.642)	0.160		
N stage (N1 & N2 & N3 vs. N0)	477	1.257 (0.960-1.647)	0.097	1.410 (0.864-2.303)	0.169
M stage (M1 vs. M0)	474	4.794 (1.765-13.016)	0.002	2.346 (0.286-19.237)	0.427
Clinical stage (Stage III & Stage IV vs. Stage I & Stage II)	485	1.214 (0.875-1.683)	0.245		
Age (> 60 vs. ≤ 60 years)	499	1.238 (0.945-1.621)	0.122		
Gender (Male vs. Female)	499	0.754 (0.566-1.004)	0.054	0.911 (0.565-1.469)	0.701
Histologic grade (G3 & G4 vs. G1 & G2)	480	0.942 (0.690-1.287)	0.709		
Radiation therapy (Yes vs. No)	438	0.623 (0.459-0.846)	0.002	0.479 (0.293-0.784)	0.003
Primary therapy outcome (CR vs. PD, SD, PR)	415	0.180 (0.122-0.264)	< 0.001	0.270 (0.156-0.465)	< 0.001
Smoker (Yes vs. No)	489	1.085 (0.775-1.520)	0.633		
Alcohol history (Yes vs. No)	488	0.967 (0.727-1.288)	0.821		
Lymphovascular invasion (Yes vs. No)	338	1.688 (1.201-2.371)	0.003	1.681 (1.044-2.707)	0.032
Lymph node neck dissection (Yes vs. No)	496	0.728 (0.524-1.012)	0.059	0.792 (0.341-1.839)	0.587
Race (White vs. Asian & Black or African American)	483	0.677 (0.448-1.023)	0.064	0.823 (0.426-1.589)	0.561
*TP53* status (Mut vs. WT)	494	1.531 (1.119-2.094)	0.008	1.223 (0.742-2.014)	0.430
*PIK3CA* status (Mut vs. WT)	494	0.988 (0.702-1.392)	0.946		
*SEC61G* (High vs. Low)	499	1.947 (1.482-2.559)	< 0.001	1.690 (1.063-2.687)	0.027

HR: hazard ratio; CI: confidence interval; CR: complete response; PD: progressive disease; SD: stable disease; PR: partial response; Mut: mutation; WT: wild type.

## Abbreviations

aDCs: activated dendritic cells
AUC: area under the curve
C-index: concordance index
DCs: dendritic cells
DEG: differentially expressed gene
iDCs: immature dendritic cells
FC: fold change
FDR: false discovery rate
GO: gene ontology
GSEA: gene set enrichment analysis
HNSCC: head and neck squamous cell carcinomas
NK cell: natural killer cell
OS: overall survival
pDCs: plasmacytoid dendritic cells
RNA-seq: RNA-sequencing
ROC: receiver operating characteristic
ssGSEA: single-sample gene set enrichment analysis
TCGA: The Cancer Genome Atlas
Tcm: central memory T cells
Tems: effector memory T cells
Tgd: gamma delta T cells
Th1: type-1 T helper cells
Th2: type-2 T helper cells
Th17: type-17 T helper cells
TPM: transcripts per million
Tregs: regulatory T cells
