# Supplementary material for: High SEC61G expression predicts poor prognosis in patients with Head and Neck Squamous Cell Carcinomas
Source: J Cancer. 2021 May 5;12(13):3887–99. doi: 10.7150/jca.51467 (PMC8176234; doi:10.7150/jca.51467)
Supplement: Supplementary file 1 — Supplementary figures. [file jcav12p3887s1.pdf]

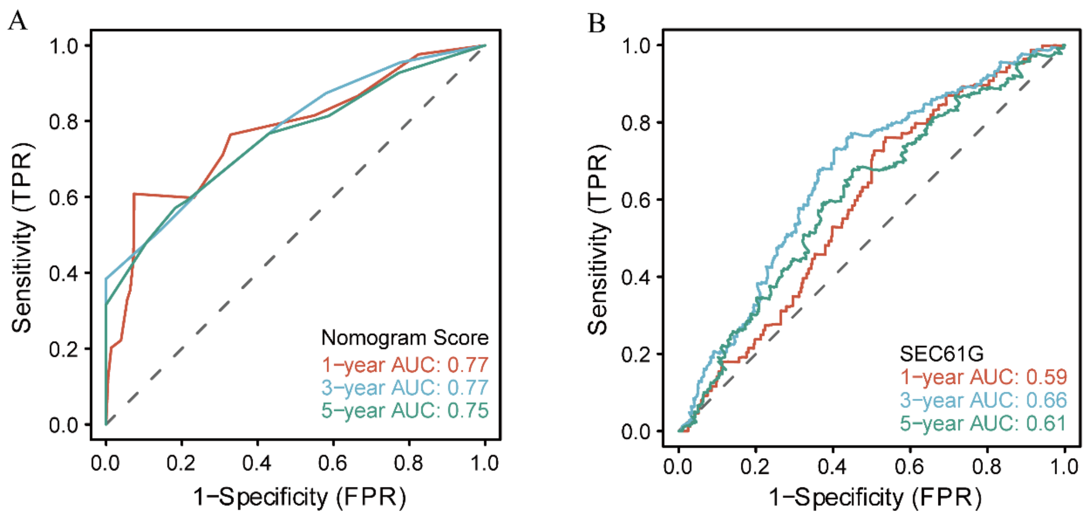

Figure S1. Time-dependent ROC analysis. Time-dependent ROCs for predicting the probability of 1-, 3-, and 5-year OS for HNSCC patients based on nomogram score (A) and SEC61G expression (B).

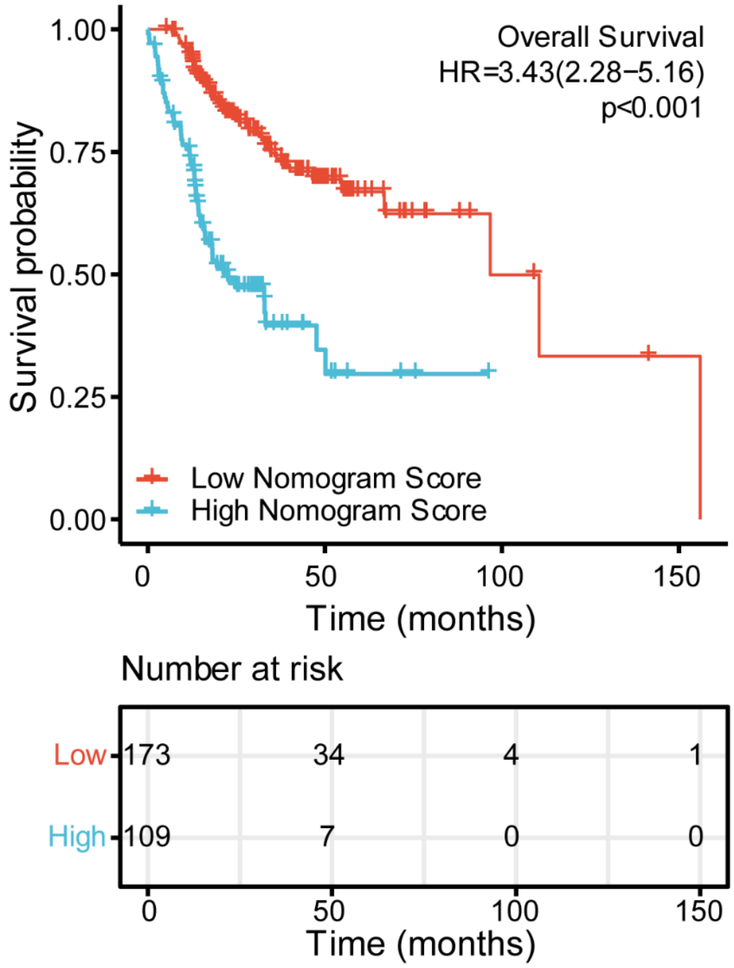

Figure S2. Kaplan–Meier curve for overall survival in HNSCC patients by nomogram score.
